# Clinical Significance of Plasma Osteopontin Level as a Biomarker of Hepatocellular Carcinoma

**DOI:** 10.4021/gr499w

**Published:** 2013-10-31

**Authors:** Mona Salem, Sahar Abdel Atti, Maisa El Raziky, Samar Kamal Darweesh, Marwa El Sharkawy

**Affiliations:** a Clinical Pathology Dept., Faculty Of Medicine, Cairo University, Cairo, Egypt; b Tropical Medicine and Hepatology Dept., Faculty Of Medicine, Cairo University, Cairo, Egypt

**Keywords:** Osteopontin, Alphafetoprotein, Hepatocellular carcinoma, Tumor marker

## Abstract

**Background:**

Biomarkers of hepatocellular carcinoma (HCC) are helpful in screening, diagnosis and follow up of cases. Osteopontin (OPN) is a glycoprotein secreted by osteoblasts, osteoclasts, macrophages and T cells, and is over-expressed in a variety of tumors, including carcinomas of liver, stomach, breast**,** lung, colon**,** and prostate. So, the aim of this study was to verify the possibility of using the plasma Osteopontin level as a biomarker for diagnosis of HCC.

**Methods:**

The study included 70 subjects divided into three groups: group I had 30 patients with HCC (proved by histopathology or combined spiral CT and elevated alpha-fetoprotein) on top of HCV, group II had 30 patients with HCV infection and group III had 10 healthy subjects serving as control. Osteopontin level was measured in plasma of the studied subjects by ELISA, serum alpha fetoprotein (AFP) level was also measured by EIA.

**Results:**

Osteopontin levels were significantly elevated in patients with HCC and in HCV patients in comparison to control group (P: 0.005). There was significant correlation between OPN and AFP levels (P: 0.00). The sensitivity and specificity of OPN for selective detection of HCC group over the non-HCC group (HCV group and healthy control group) were73% and 54%, respectively, at a cut-off value of 128.5 ng/mL. Plasma OPN levels directly correlated with the tumor number but not with the size of the tumor (P: 0.00).

**Conclusion:**

Plasma OPN level appears to be an additional biomarker for HCC detection.

## Introduction

HCC is the most common cause of primary liver neoplasms and the fourth most frequent type of cancer worldwide following lung, breast and bowel cancers with an increasing incidence, causing one million deaths per year [1].

Screening strategies including alpha-fetoprotein (AFP) and ultrasound every 6 months in patients with liver cirrhosis have been recommended to detect HCC at earlier stages leading to effective treatment strategies. AFP, however, is a marker with poor sensitivity and specificity and ultrasound is highly dependent on the operator’s experience [2]. So, the use of cancer biomarkers to anticipate the outlines of disease has been an emerging issue, especially as cancer treatment has made such positive steps in the last few years [3].

Osteopontin (OPN) is a phosphorylated glycoprotein secreted by activated macrophages, leukocytes, and activated T lymphocytes. Over-expression of OPN has been found in a variety of cancers, including carcinomas of stomach [4], breast [5], prostate [6], lung [7], colon [8], and liver [9].

OPN over-expression tended to be associated with the presence of tumor vascular invasion and advanced tumor grade, thus, indicating poor prognosis for patients with HCC, it may also have predictive potential for HCC invasion and metastasis [10]. Also it was found that Interference of osteopontin expression inhibits the invasion and metastasis of human hepatocellular carcinoma [11], this opens the potential for OPN directed treatment that could greatly enhance outcomes for HCC patients.

The aim of this work was to verify the possibility of using the plasma Osteopontin level as a potential biomarker for hepatocellular carcinoma.

## Patients and Methods

This study was conducted on 60 patients (after approval of the ethical committee); they were selected from the Tropical Medicine Department, Cairo University and 10 healthy subjects as control group. The study was carried out in accordance with The Code of Ethics of the World Medical Association (Declaration of Helsinki) for experiments involving humans. Patients (aged 40 to 70 years old) were divided as follow: Group I: 30 patients with hepatocellular carcinomas (proved by histopathology or combined triphasic CT and elevated alpha-fetoprotein) on top of hepatitis C virus infection as diagnosed by seropositivity for HCV antibodies; Group II: 30 patients with HCV infection as diagnosed by seropositivity for HCV antibodies; Group III: 10 apparently healthy subjects, age and sex matched, having no acute or chronic illness and taking no medications, were included as control group.

Exclusion criteria were: (1) Patients with any other tumor than HCC; (2) Patients with metastases of HCC; (3) Patients with other chronic liver diseases (for example, HBV); (4) Patients with bony lesions or inflammatory diseases and (5) Patients with poorly controlled diabetes mellitus or systemic hypertension.

All patients and controls (after informed consent) were subjected to: (A) History taking, (B) Liver and other biochemical profiles including AST, ALT, serum albumin, total billirubin, Prothrombin time, creatinine, and CBC. All were assayed using Hitachi auto analyzer and the kits were supplied from Roche Diagnostic, Germany. (C) Abdominal ultrasound with special emphasis on liver, focal hepatic lesions, spleen and presence or absence of ascites. (D) Serum Alpha Fetoprotein assayed using enzymatic immunochemiluminesent using Immulite (Semeins). (E) Plasma Osteopontin level measuring (F) CT abdomen and chest with bone scan (done for patients only) to exclude metastases of HCC.

### Plasma osteopontin assay

Plasma Osteopontin (OPN) was measured by enzyme linked immunosorbent assay (ELISA) using recombinant human OPN ELISA. R&D Systems, Inc. 614 McKinley Place NE Minneapolis, MN 55413 United States of America.

### Serum alpha fetoprotein assay

Serum Alpha fetoprotein was measured by human AFP EIA kit lot. REF 600-10 manufactured by CanAg Diagnostics AB, Majnabble Terminal SE-414 55 Gothenburg, and Sweden.

### Statistical methods

The SPSS 10.0 for windows was used for data management and analysis and the Microsoft PowerPoint for charts. Quantitative data were presented as mean ± SD. For comparison of the two groups’ means, the Student’s t-test was used, while for the comparison of the three groups’ means, one way analysis of variance (ANOVA) was used followed by Post Hoc test. Non parametric quantitative data were expressed as median (range), Kruskall-wallis and Mann-whitney tests were used for comparison of means. Qualitative data was expressed as frequency and percentage. Association between qualitative data was done using Chi- square test. Risk estimate was done by odds ratio. P value was considered significant at 0.05.

## Results

Group I included 21 males and 9 females with a mean age 56.7 ± 8.9 years while group II included 25 males and 5 females with a mean age 51 ± 12 years.

Fatigue was the most common clinical presentation in group I (96.7%), while jaundice was the most common clinical presentation in group II (30%).

By US, The frequencies of hepatomegaly, cirrhosis, splenomegaly and ascites were 70%, 93.3%, 70% and 83.3% in group I, and 83.3%, 96.7%, 50% and 50% in group II respectively. HCC were detected in the right lobe in 17 (56.7%) patients, in the left lobe in 10 (33.3%) patients and in both lobes in 3 (10%). Lesions were < 3 cm in 12 (40%) and ≥ 3 cm in 18 (60%). Nineteen (63.3%) patients had single lesion and 11 (36.7%) had multiple lesions.

Plasma OPN levels were not significantly affected by sex (in all three groups) or by hematemsis or size of the tumor (in groups I and II). But, OPN levels in the HCC group were affected by the presence of cirrhosis and ascites. We also observed an increase of plasma OPN levels depending on the tumor multiplicity (Table 1). Also, OPN showed direct significant correlation with the age of the patients, AST and ALT levels. While it showed inverse significant correlation with albumin, total billirubin, PC and platelets (Table 2).

**Table 1:** OPN Levels of all the Studied Patients Versus Various Clinical Parameters (n = 60)

Clinico-pathological features	No	Osteopontin ng/dL	P-value
Sex			
male	52	136 (79 - 189)	0.55
female	18	104 (54 - 221)	
Hematem	5	178 (65 - 560)	0.54
Cirrhosis	57	148 (90 - 207)	0.00
Ascites	40	171 (115 - 303)	0.00
Liver	1		
Enlarged	36	140 (83 - 193)	0.001
Normal	1	39 (33 - 44)	
Tumor Size			
< 3 cm	12	140 (100 - 336)	0.28
≥ 3 cm	8	229 (131 - 438)	
Multiplicity			
Single	19	138 (97 - 178)	0.00
Multiple	11	495 (388 - 717)	

**Table 2:** Correlations Between OPN and AFP and Other Parameters for all Studied Patients

	OPN		AFP	
	r	P	r	P
Age (y)	0.448	0.000	0.476	0.000
AST (IU/L)	0.455	0.000	0.435	0.000
ALT (IU/L)	0.328	0.006	0.171	0.158
Alb (g/dL)	-0.614	0.000	-0.274	0.017
T.Bil. (mg/dL)	-.452	0.000	0.282	0.018
PC%	-0.33	0.005	-0.31	0.009
Hb (gm/dL)	-0.13	0.268	-0.07	0.95
Plat. (× 10^3^/mm^3^)	-0.33	0.004	-0.43	0.000
AFP (ng/mL)	0.408	0.000		

AFP showed direct significant correlation with the age of the patients, AST and total billirubin level. It also showed inverse significant correlation with albumin, PC and platelets (Table 2).

**Figure 1:**
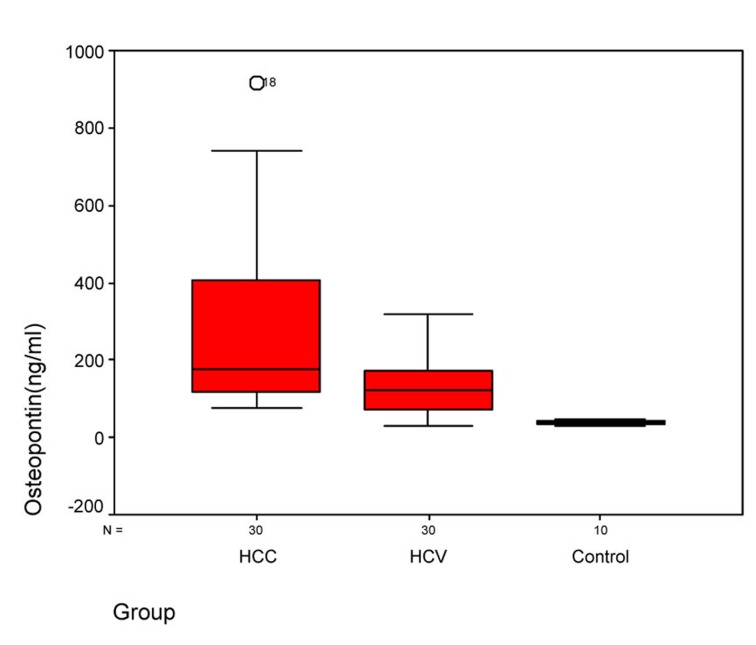
Figure 1. Plasma osteopontin levels in three groups. Box plots represent median, quartiles and extremes, the asterisks represent outliers.

**Figure 2:**
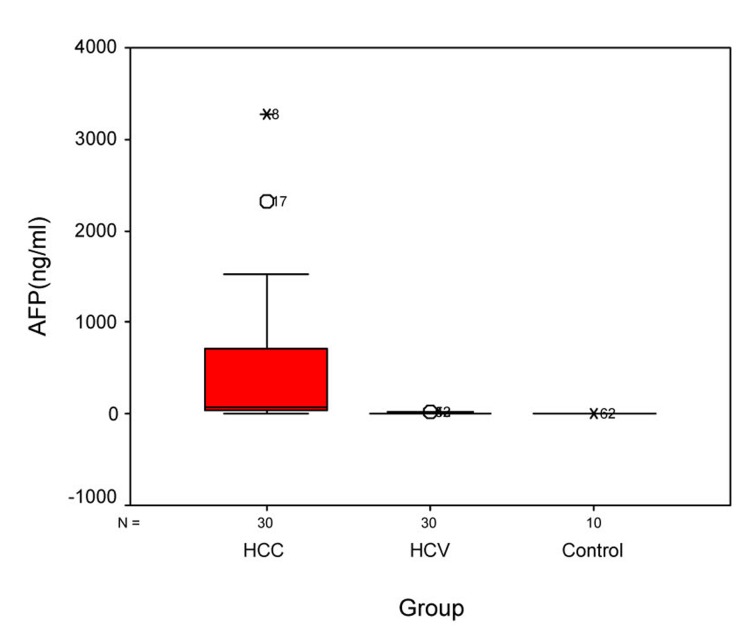
Figure 2. Serum AFP levels in different groups. Box plots represent median, quartiles and extremes, the asterisks represent outliers.

Statistical analysis of OPN and AFP levels in the 3 groups showed that there was significant difference between group I and group II, between group II and control group, and between group I and control group (P ≤ 0.005) (Table 3). Statistical analysis of the OPN showed that the median plasma OPN level was significantly higher in the HCC group than in the HCV group or in the normal control group as determined by Mann-Whitney U test (P-value < 0.001) (Fig. 1). Also, the median serum AFP level was higher in the HCC group than in the HCV group or in control group as determined by Mann-Whitney U test (P-value < 0.001) (Fig. 2).

**Table 3:** Comparison of Alpha Fetoprotein and Osteopontin in the 3 Groups

	Group I (n = 30)	Group II (n = 30)	Control (n = 10)	P value
AFP (ng/mL)	79 (38 - 726) a	5.5 (4 - 10) b	3.5 (3 - 4.4) c	0.00*
OPN (ng/mL)	178 (116 - 410) a	122.5 (73 - 173) b	37.5 (33 - 44) c	0.00*

* P-value calculated by Kruskal-Wallis Test. P-value is significant if < 0.05. Groups having the same letters are not statistically significance.

**Figure 3:**
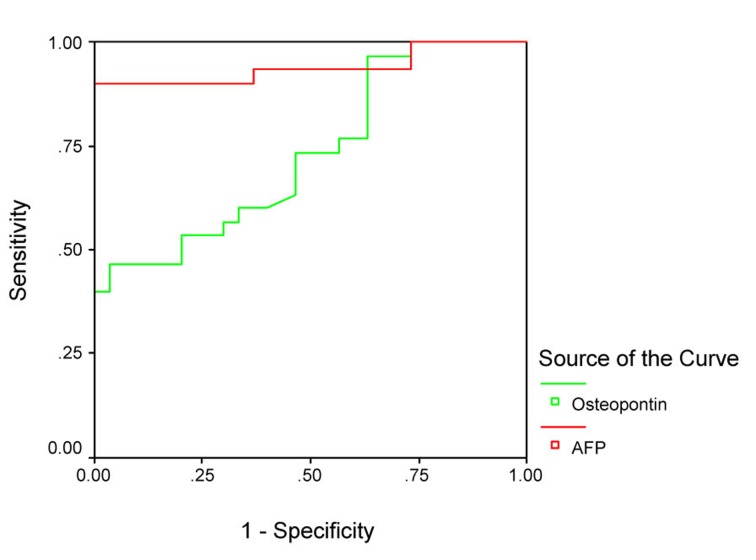
Receiver operating characteristics (ROC) curve analysis of plasma OPN in comparison with AFP for discrimination between groups 1 and the other 2 groups.

**Figure 4:**
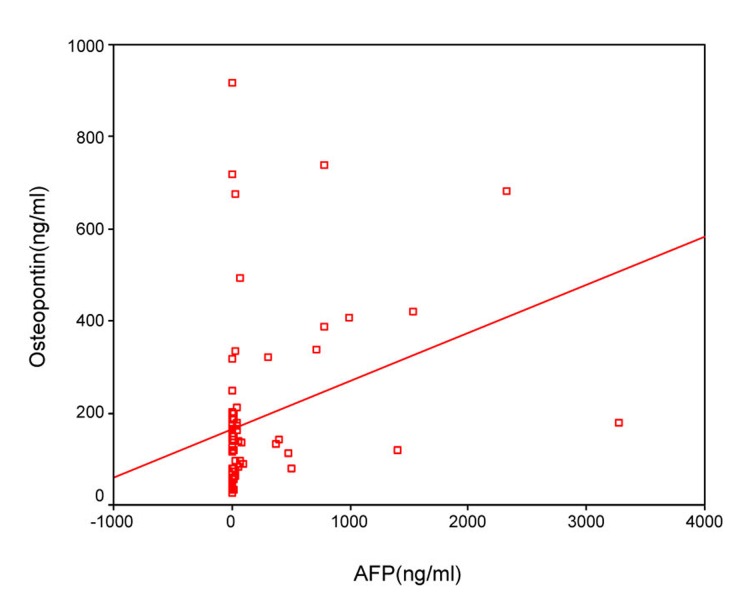
Direct significant correlation between plasma OPN and serum AFP level of the patient as serum AFP level increases with increase of osteopontin level.

The sensitivity and specificity of OPN for selective detection of the HCC group over the non-HCC group (HCV group and healthy control group) were 73% and 54%, respectively; at a cut-off value 128.5 ng/mL. While the sensitivity and specificity of AFP for selective detection of the HCC group over the non-HCC group were 90% and 77%, respectively, at a cut-off value 10.4 ng/mL (Table 4, Fig. 3). There was direct significant correlation between plasma OPN and serum AFP level of patients as serum AFP level increases with increase of osteopontin level (Fig. 4).

**Table 4:** Diagnostic Sensitivity and Specificity of Plasma OPN Level of HCC Patients in Comparison With AFP for Selective Detection of HCC

	Sensitivity (%)	Specificity (%)	Cut-off (ng/mL)
OPN	73%	54%	128.5
AFP	90%	77%	10.4

## Discussion

Early detection of patients with HCC is an attractive goal because it gives better prognosis as HCC tends to grow slowly and stay confined to the liver. Early detection is possible with ultrasound scanning and AFP monitoring, although the use of AFP as a screening test is complicated by frequent false positive and false negative results [12], so early diagnosis of HCC would not be difficult if tumor markers and medical imaging were combined [13].

Also, early detection of HCC opens doors for various effective treatments such as surgical resection, radiofrequency ablation, and transplantation, which can subsequently lead to long-term survivals in a great number of HCC patients [14].

In our study, all patients were selected to have anti-HCV sero-positivity due to its high prevalence rate which was reported by Nair et al [15]. Also Soliman [16] found that 84.2% of HCC patients were HCV positive.

A total of 93.3% of patients, in our study, were cirrhotic and this was the major clinical risk factor for HCC development, this was in agreement with Blum [17], who reported that 90% of patients developed HCC on top of cirrhosis, and with Dobrila et al [18] who found underlying cirrhosis in 80.55% patients with HCC.

The sex of patients, in our study, showed statistically significant difference in HCC patients with a male: female ratio 2: 1, this male predominance was also observed by Goldman and Ausiello [19], who reported a male: female ratio 2:1 up to 4:1. And also reported by Egyptian studies done by Nabeel [20] and Lehman et al [21].

In our study, the age of HCC patients ranged from 39 to 70 years with a mean of 56.7 ± 8.9 years and this is probably attributed to the duration of the underlying liver disease, also Di Bsiceglie [22] stated that HCC is reported to develop in the fifth decade. The same results were reported by Johnson [23], who found that the average age of patients ranged from fifth to sixth decades of life.

By ultrasound examination, most of the HCC lesions (56.7%), in our study, were found in the right lobe of the liver. Similarly, Rosen and Nogarney [24] documented that HCC occurs most frequently in the right lobe of the liver either as a solitary mass or as multiple nodules. This may be due to the large size of the right lobe of the liver which is 6 times the left lobe [25]. Also, Haseeb [26] and ElKady et al [27] found in their study that 75-100% of focal lesions were found in the right lobe.

The sensitivity and specificity of AFP has been shown to vary with the different cutoff values used. According to our results, at a cutoff 10.4 ng/mL the sensitivity was 90% and the specificity was 77%. These results were comparable to those of Taketa et al [28] who reported sensitivity 95% and specificity 66% with the cutoff value 10 ng/mL, which is the cutoff level of healthy subjects. Gad et al [29] found a significantly higher sensitivity of AFP in Egyptian patients in comparison with Japanese patients for HCC diagnosis (99 % versus 67% P < 0.001) for AFP level greater than 10 ng/mL, with comparable specificity (75% versus 82%).

A significant inverse correlation was found, in our study, between AFP and number of platelets as patients with low platelet count had higher levels of serum AFP. This could be explained by the progression of liver cirrhosis with progressive decreased platelets (due to portal hypertension and splenomegaly). Similarly, Di Bisceglie et al [30] found that among patients with chronic HCV decreased platelet count is associated with elevated serum AFP level.

By studying OPN level in different groups, relation to sex of patients, relation to tumor number or size, sensitivity, specificity and relation to AFP level, we found that: Significant elevation of plasma osteopontin levels in HCC patients than HCV patients’ levels and lower levels in normal control group was evident in our study. Kim et al [14] and Zhao et al [31] also found that median plasma OPN level in HCC patients was 955 (range 168 - 5,742 ng/mL) and 13.38 (range 9.2 - 23.6 ng/mL) respectively, and in CLD was 381 (range 29 - 1,688 ng/mL) and 4.5 (range 3.15 - 6.43 ng/mL) respectively. Also, Zhang et al [32], El-Din Bessa et al [33] and Abu El Makarem et al [34] found that the median plasma OPN level was significantly higher in the HCC group than in the cirrhotic patients or in the normal control group.

OPN levels were not significantly affected by sex of patients in our study; these results were similar to those of Kim et al [14] and Zhao et al [31] as both found no differences in OPN levels between males and females.

The median plasma OPN level, in our patients, with single tumor nodule was 138 ng/mL, while in patients with multiple tumor nodules was 495 ng/mL and this was statistically significant (P value: 0.00). This corresponded to the results of Zhang et al [32], as they found that the median plasma OPN level of patients with multiple tumor nodules (217.11 ng/mL) was higher than that of patients with a single tumor nodule (168.18 ng/mL).

However, there was no significant correlation between OPN level and tumor size in our study, as tumors < 3 cm, present in 40% of patients, showed median plasma OPN level 140 with a range of (100 - 336 ng/mL), and tumors ≥ 3 cm, present in 60% of patients, showed median plasma OPN level 229 with a range of (131 - 438 ng/mL) (P value: 0.28). The relation between OPN and tumor size was also studied by Zhang et al [32], and they found that tumors ≤ 5 cm showed median plasma OPN level 176.90 ng/mL, and tumors > 5 cm showed median plasma OPN level 172.92 ng/mL.

The sensitivity and specificity of OPN has been shown to vary with the different cutoff values used. According to our results, the sensitivity and specificity of OPN for selective detection of the HCC group over the non-HCC group were comparable to those of Kim et al [14] who reported diagnostic sensitivity and specificity of OPN for HCC group over non-HCC group (CLD group and healthy control) to be 93.5% and 84.2%, respectively, at a cut-off level of 552.9 ng/mL.

From the results of the study of Matsui et al [35], it is suggested that 200 ng/mL could be set as the critical cut-off point for predicting the prognosis of patients with HCC. However, the reported normal median plasma OPN levels are highly variable, with a range from 31 ng/mL to even > 200 ng/mL. The exact reason for this difference is not very clear, but it might be due to the different assay systems and conditions of sample collection used in those studies.

Another important issue that limits the potential utility of blood OPN levels as a specific biomarker for cancer is that OPN level is also increased in a range of inflammatory syndromes [35]. Therefore, further careful evaluation with one standardized assay system will be needed to gain greater insight into the potential usefulness of OPN in patients with HCC.

Our results showed that there was significant positive correlation between OPN and AFP levels and similarly Zhang et al [32] found that the plasma OPN level positively correlated with the serum AFP concentration. However, Kim et al [14] found that the correlation between plasma OPN and serum AFP levels was insignificant.

### Conclusion

The results obtained in this study are valuable for the future application of plasma OPN level as a routine biomarker for the diagnosis and clinical prediction of recurrence, metastasis, and prognosis in patients with HCC.

## Abbreviations

OPN: Osteopontin
HCC: Hepatocellular carcinoma
AFP: alphafetoprotein
